# Difference in intracellular temperature rise between matured and precursor brown adipocytes in response to uncoupler and β-adrenergic agonist stimuli

**DOI:** 10.1038/s41598-017-12634-7

**Published:** 2017-10-10

**Authors:** Toshikazu Tsuji, Kumiko Ikado, Hideki Koizumi, Seiichi Uchiyama, Kazuaki Kajimoto

**Affiliations:** 1 Central Laboratories for Key Technologies, KIRIN Company Limited, 1-13-5 Fukuura, Kanazawa-ku, Yokohama-shi, Kanagawa 236-0004 Japan; 2 Graduate School of Pharmaceutical Sciences, The University of Tokyo, 7-3-1 Hongo, Bunkyo-ku, Tokyo 113-0033 Japan; 3 Faculty of Pharmaceutical Sciences, Hokkaido University, Kita-12, Nishi-6, Kita-ku, Sapporo, Hokkaido 060–0812 Japan; 4 Present Address: Health Research Institute, National Institute of Advanced Industrial Science and Technology (AIST), 2217-14, Hayashi-cho, Takamatsu, Kagawa 761-0395 Japan

## Abstract

Brown adipocytes function to maintain body temperature by heat production. However, direct measurement of heat production at a single cell level remains difficult. Here we developed a method to measure the temperature within primary cultured brown adipocytes using a cationic fluorescent polymeric thermometer. Placement of the thermometer within a matured brown adipocyte and a precursor cell enabled the detection of heat production following uncoupler treatment. The increase in the intracellular temperature due to stimulation with a mitochondrial uncoupler was higher in matured brown adipocytes than in precursor cells. Stimulation with a β-adrenergic receptor (β-AR) agonist, norepinephrine, raised the intracellular temperature of matured brown adipocytes to a level comparable to that observed after stimulation with a β3-AR-specific agonist, CL316.243. In contrast, neither β-AR agonist induced an intracellular temperature increase in precursor cells. Further, pretreatment of brown adipocytes with a β3-AR antagonist inhibited the norepinephrine-stimulated elevation of temperature. These results demonstrate that our novel method successfully determined the difference in intracellular temperature increase between matured brown adipocytes and precursor cells in response to stimulation by an uncoupler and β-AR agonists.

## Introduction

In mammals, brown adipose cells (BACs) play a major role both in maintaining a stable body temperature through non-shivering thermogenesis and in regulating whole-body energy expenditure^1,2^. While most other tissues produce heat at only 1 W/kg, brown adipose tissue (BAT) can generate heat at up to 300 W/kg^3^. Recent studies have focused on strategies to increase BAT activity as a potential approach for treating obesity and related metabolic disorders, which are becoming severe global public health concerns. For example, previous reports showed that the transplantation of fibroblasts expressing PRDM16 and C/EBP-β or human induced pluripotent stem cells transferring exogenous genes (PPARG2, CEBPB and PRDM16) into mice engendered active BAT with high glucose uptake^4,5^. The transplantation of functional BACs developed from human-induced pluripotent stem cells without gene transfer into mice was found to improve the symptoms of obesity through augmented glucose and lipid tolerance^6^. In addition, food science studies have shown that food ingredients, such as capsaicin, fish oil, Thai black ginger extract and hop extract, can induce BAT activity and increase energy expenditure by stimulating the sympathetic nervous system^7–10^.

BAT thermogenesis is regulated by norepinephrine (NE) released from sympathetic nerve terminals. NE activates β-adrenergic receptors (β-ARs), leading to an accumulation of intracellular cAMP and induction of hormone-sensitive lipase to hydrolyze intracellular triglycerides. The fatty acids released from triglycerides then activate uncoupling protein 1 (UCP1), a BAC-specific molecular marker located in the mitochondrial inner membrane^11^, that functions to uncouple the electrochemical proton gradient to dissipate the energy for heat generation.

Extensive studies have determined the critical transcriptional regulator and signaling pathways involved in BAC differentiation. In recent years, a novel type of BAC, called beige or brite adipocyte, that is interspersed in white adipose tissue has received much attention^12,13^. Chronic cold exposure and stimulation with β3-AR agonists activates these beige/brite cells in adults^14^. White adipose tissue-derived multipotent stem cells transplanted into mice with original hydrogels could differentiate into beige cells, resulting in enhancement of respiration rates and reduction of weight gain of implanted mice^15^. However, the precise molecular mechanisms and signaling pathways that control brown or beige/brite adipocyte differentiation are largely unknown.

Despite the number of studies on the differentiation of BACs, precise methods to evaluate BAC function, such as determining UCP1 protein expression, BAC-specific gene expression, or oxygen consumption, remain limited. Thus, it has been difficult to assess how a gene of interest in BACs might affect thermogenesis. A few approaches to directly assess thermogenesis have been reported, such as calorimetry using whole BACs^16^ and isolated mitochondria^17^. However, these techniques require many cells because of the low sensitivity of the measuring devices. Moreover, the main heat source is within the cell. Thus, a method to detect intracellular thermogenesis directly, such as an intracellular thermometer, is required.

Recently, several fluorescence-based intracellular thermometry probes have been developed with high-temperature resolution (better than 1 °C) and high spatial resolution (molecular scale in principle), including a temperature-sensitive polymer^18–20^, temperature-sensitive gel^21,22^, quantum dots^23–25^, green fluorescent protein^26–28^, europium complex-containing nanoparticles^29^, molecular beacon^30^, gold nanocluster^31^, nanodiamond^32^ and organic small molecules^33,34^. We previously developed a cationic fluorescent polymeric thermometer (CFPT) with the ability to penetrate living cells and perform intracellular temperature measurements in yeast and mammalian cells^19,20^. The advantages of CFPT^18,20^ compared with other thermometers include high accuracy in detecting a temperature change of 0.1 °C and spontaneous and rapid incorporation into cells (cytoplasm and nucleus) without special techniques (see the comparison of thermometers in Table S1). Recently, a CFPT with two fluorophores for ratiometric sensing, called an R-CFPT^35^, was developed to clarify the precise temperature change with fluorescence intensity and to improve temporal resolution compared with fluorescence lifetime measurements^18,20^.

**Figure 1:**
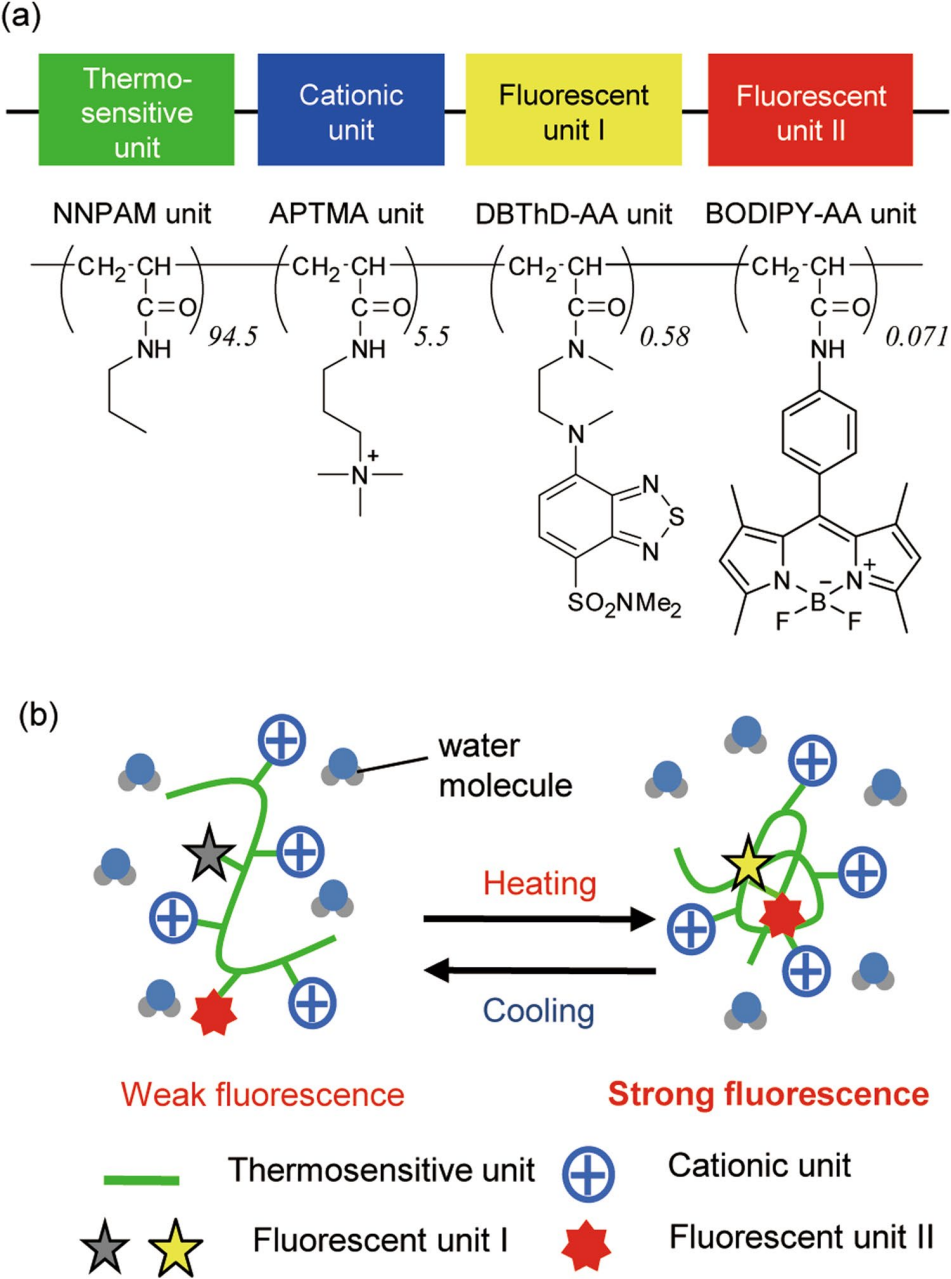
Cationic fluorescent polymeric thermometer (R-CFPT) for intracellular thermometry. (**a**) Chemical structure of R-CFPT. Mw = 9260, Mn = 4540, Mw/Mn = 2.0. NNPAM, *N*-*n*-propylacrylamide; APTMA, (3-acrylamidopropyl)trimethylammonium; DBThD-AA, *N*-(2-{[7-(*N*,*N*-dimethylaminosulfonyl)-2,1,3-benzothiadiazol-4-yl]-(methyl)amino}ethyl)-*N*-methylacrylamide; BODIPY-AA, 8-(4-acrylamidophenyl)-4,4-difluoro-1,3,5,7-tetramethyl-4-bora-*3a,4a*-diaza-*s*-indacene. (**b**) Functional diagram of R-CFPT in aqueous medium. At low temperature, the thermo-responsive polyNNPAM sequence in R-CFPT (green line) assumes an extended structure such that the water-sensitive DBThD-AA unit (gray star) can be quenched by neighboring water molecules. At higher temperature, the polyNNPAM sequence shrinks because of hydrophobic interactions among the NNPAM units (green line), resulting in the release of water molecules and strong fluorescence from the DBThD-AA unit (yellow star). The fluorescence intensity of the second fluorescent unit, BODIPY-AA (red star), is relatively insensitive to temperature-dependent changes in the polyNNPAM structure. The cationic APTMA unit (blue symbol) promotes incorporation of R-CFPT into the cell and enriches its hydrophilicity to prevent interpolymeric aggregation within a cell.

R-CFPT is a random copolymer composed of four distinct units: a thermosensitive unit, a cationic unit and two fluorescent units (Fig. 1a). At low temperatures, the thermo-sensitive polyNNPAM sequence in the R-CFPT assumes an extended structure such that neighboring water molecules can quench the water-sensitive fluorescent DBThD-AA unit (Fig. 1b)^36^. At higher temperatures, the polyNNPAM sequence shrinks because of hydrophobic interactions among the NNPAM units, resulting in the release of water molecules and strong fluorescence from the DBThD-AA unit. In addition, the fluorescence intensity of the second fluorescent unit, BODIPY-AA^35^, is relatively insensitive to temperature-dependent changes in the polyNNPAM structure; in other words, it emits constant fluorescence as a reference signal. The cationic APTMA unit promotes incorporation of R-CFPT into a cell^19^ and enriches its hydrophilicity to prevent interpolymeric aggregation within a cell^37^. The fluorescence response of R-CFPT to temperature variation is independent of the concentration of R-CFPT, the ionic strength (0.25–0.35) and the environmental pH in the range of 6–9^35^.

Three thermometers, a modified GFP^27^, a polymeric nanogel^22^ and a BODIPY-based organic dye^34^, have been previously used to measure the intracellular temperature of BACs. However, each of these thermometers shows limitations. The modified GFP showed a lower sensitivity compared with the polymeric nanogel, BODIPY-based organic dye, and R-CFPT. The polymeric nanogel requires microinjection to deliver it into the cell, and the BODIPY-based organic dye can only detect endoplasmic reticulum (ER) temperature in BACs. Therefore, among the current thermometers, R-CFPT has advantages in terms of high sensitivity, the ability to penetrate living cells and detection of whole cell temperature distribution.

In this study, we established a method that can measure temperature within BACs based on microscopic observation of primary cultured BACs and R-CFPT. Isidor *et al*. reported that the incorporation of siRNA (molecular weight, ~14,000) into matured adipocytes is challenging due to low efficiency^38^. Therefore, the application of polymer R-CFPT (number-average molecular weight, 10,800) into matured BACs required optimization of the procedure to introduce it into cells. Using the optimized method, we have determined differences in the intracellular temperature changes between matured BACs and pre-BACs in response to uncoupler and β-AR stimuli, including the known β-AR agonist NE and the known β3-AR-selective agonist CL316.243^39^.

## Results

### Characteristics of primary cultured BACs

**Figure 2:**
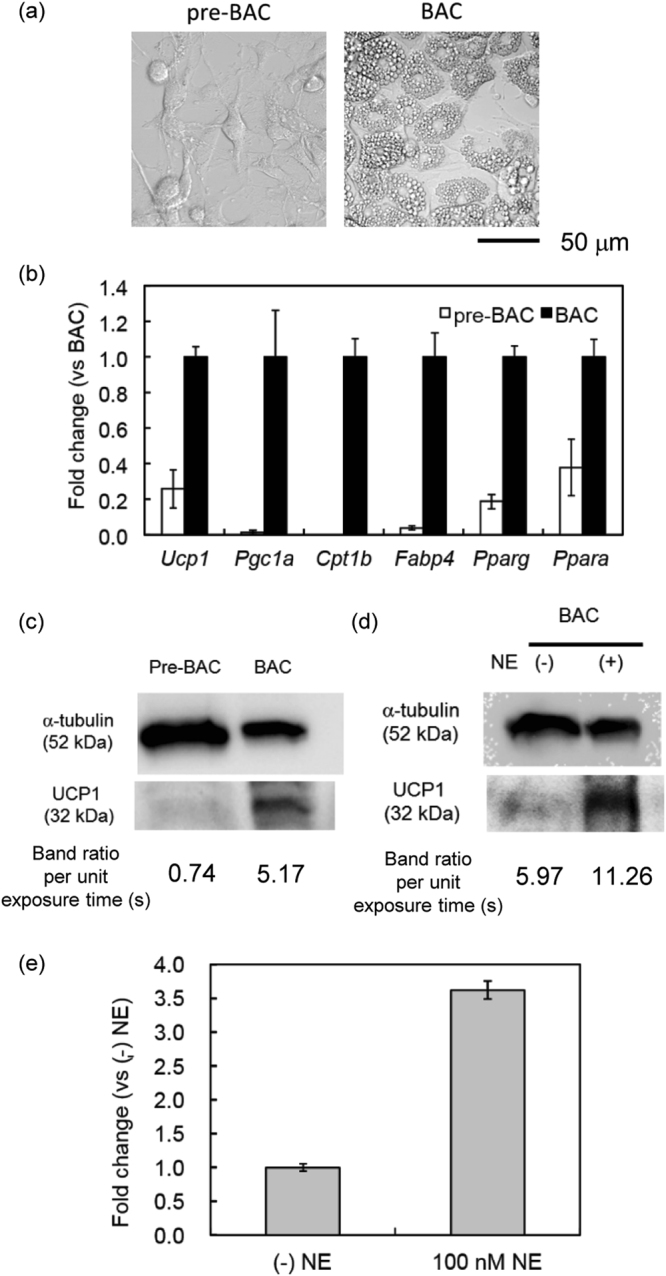
Physiological properties of primary cultured brown adipocytes (BACs) in this study. (**a**) Representative differential interference contrast (DIC) images of pre-BACs and matured BACs. Bar, 50 µm. (**b**) UCP1, PGC-1α, CPT-1β, FABP4, PPARγ, and PPARα mRNA expression in primary culture of pre-BACs and matured BACs measured by RT-PCR (*n* = 3, mean ± SD). Data were normalized to the corresponding 18S rRNA values. (**c**) Western blot analysis of UCP1 protein expression in pre-BACs and BACs (20 μg protein from cell lysate). The exposure times to detect α-tubulin and UCP1 bands were 8 s and 60 s, respectively. (**d**) UCP1 protein expression in BACs (10 μg protein) in response to NE stimulation. The exposure times to detect α-tubulin and UCP1 bands were 15 s and 240 s, respectively. (**e**) Expression of UCP1 mRNA in BACs in response to NE stimulation as measured by RT-PCR (*n* = 3, mean ± SD). Data were normalized to the corresponding 18S rRNA values.

Differentiated BACs showed multilocular morphology, whereas primary undifferentiated pre-BACs from stromal vascular cells showed a fibroblast-like morphology (Fig. 2a). Almost all cells in the differentiation culture had accumulated several lipid droplets, indicating that the differentiation had occurred with high efficiency. Browning of primary adipocytes from stromal vascular cells of interscapular BAT can be evaluated by measuring the mRNA expression of UCP1 and other gene markers of matured BACs by qRT-PCR. We selected PGC-1α as a BAC-specific gene marker, which induces mitochondrial biogenesis and UCP1 expression, resulting in thermogenesis^40^. We also measured the mRNA expression of FABP4 and PPARγ, which are major adipocyte markers, and CPT-1β and PPARα, which are known markers of BACs. As shown in Fig. 2b, the expressions  of  UCP1 and PGC-1α mRNA were much higher in matured BACs than in pre-BACs. In addition, the mRNA expression levels of CPT-1β, FABP4, PPARα, and PPARγ were at least twice as high in BACs as in pre-BACs. To confirm that the protein level of UCP1 had increased in BACs, we performed western blot analysis and confirmed that UCP1 protein expression was strongly induced by differentiation in primary culture (Figs.  2c and S1a). In addition, the stimulation of BACs by 100 nM NE induced intense expression of UCP1 gene and protein (Figs. 2d, S1b and 2e), which is an essential functional characteristic of BACs^41^.

**Figure 3:**
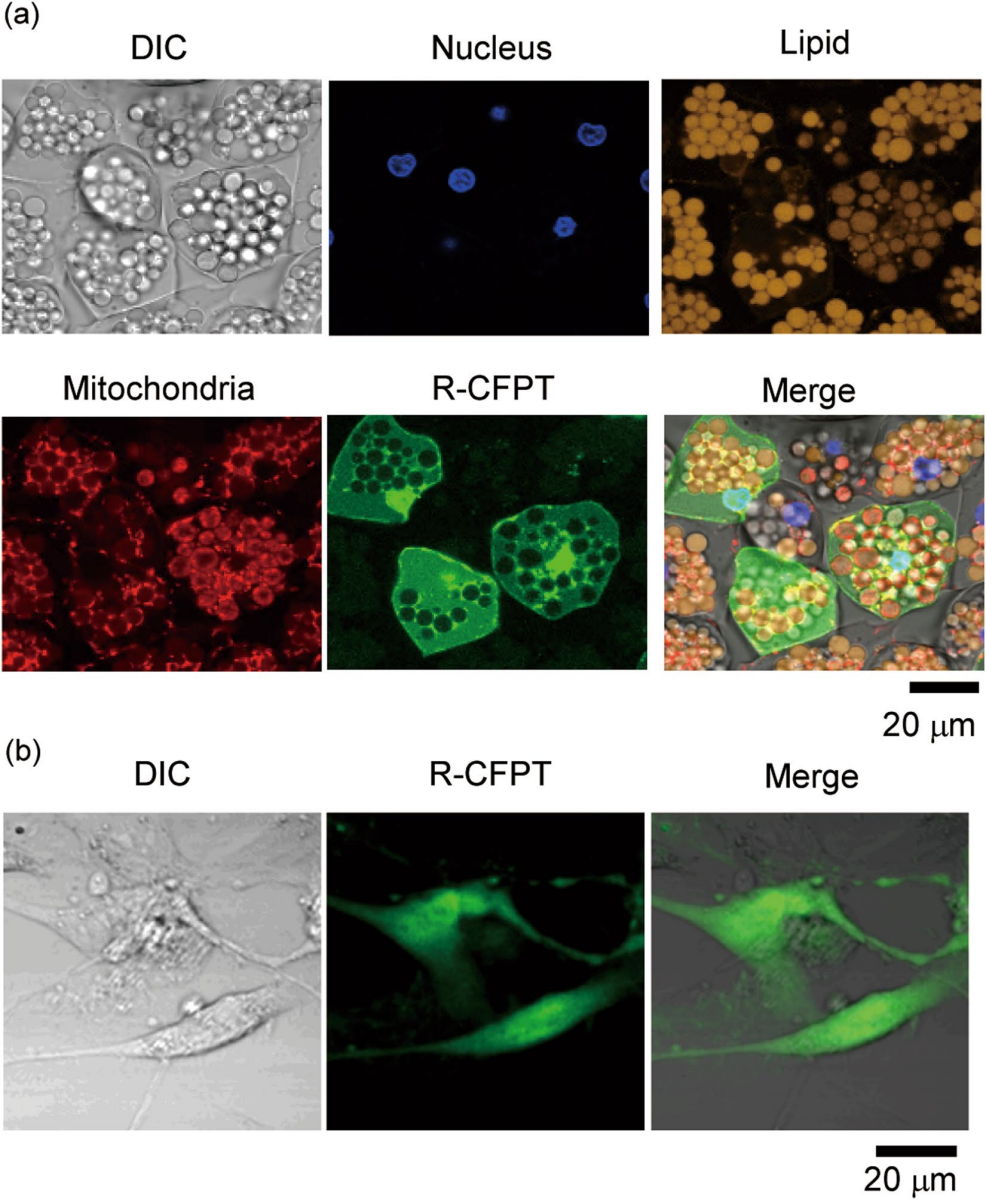
Incorporation of intracellular thermometer R-CFPT into BACs. (**a**) DIC image (upper left), nuclei staining (Hoechst33342, upper middle), lipid staining (NileRed, upper right), mitochondria staining (MitoTrackerDeepRed, lower left), confocal fluorescence image of R-CFPT (lower middle), and merged image (lower right) of BACs treated with 0.04 w/v% R-CFPT. (**b**) DIC image (left), confocal fluorescence image of R-CFPT (middle), and merged image (right) of pre-BACs treated with 0.05 w/v% R-CFPT. Bars, 20 µm.

We next introduced the R-CFPT thermometer into BACs by referring to the previously established procedure for MOLT-4 (human acute lymphoblastic leukemia) and HEK293T (human embryonic kidney) cells^35^. We treated cells with 0.05 w/v% of R-CFPT in 5% glucose solution at 25 °C for 10 min. Representative differential interference contrast (DIC) and confocal fluorescence images of R-CFPT, Hoechst33342 (nucleus), NileRed (lipid), and MitoTrackerDeepRed (mitochondria) of BACs after treatment with R-CFPT are shown in Fig. 3a. As reported for HEK293T and MOLT-4 cells^35^, R-CFPT showed spontaneous uptake into the BACs. The R-CFPT-stained area did not overlap with the NileRed-stained lipid droplets or the MitoTrackerDeepRed-stained mitochondria, indicating that R-CFPT had diffused throughout the cytosol and the nucleus.

Next, to optimize the R-CFPT incorporation efficiency and cytotoxicity, BACs were incubated with various concentrations of R-CFPT (0.01, 0.03, 0.04 or 0.05 w/v%) in 5 w/v% glucose at 25 °C for 10 min, and the incorporation efficiency of R-CFPT was calculated from confocal fluorescence images taken at the various R-CFPT concentrations. As shown in Table 1, the incorporation efficiency increased as the relative proportion of R-CFPT rose from 0.01 to 0.05 w/v%. Cytotoxicity was assessed by propidium iodide (PI) assay, which is generally used to estimate damage to the plasma membrane and serves as a marker of cell death^42^. Cytotoxicity was determined in cells cultured for 30 min after introduction of R-CFPT. As shown in Table 1, there was no significant difference in cell viability between non-R-CFPT treated cells and cells treated with R-CFPT at a concentration of 0.04 w/v% or lower. At 0.05 w/v% R-CFPT, some cells began to die, as evidenced by plasma membrane rupture (Table 1). Based on these results, we chose 0.04 w/v% R-CFPT for subsequent experiments to measure intracellular temperature, because this concentration showed sufficient cellular incorporation efficiency and almost no cytotoxicity.

**Table 1:** Effect of R-CFPT concentration on incorporation efficiency and cytotoxicity in BACs.

R-CFPT concentration (w/v%)	Incorporation efficiency (%)^a^	Viability of cells (%)^a^
0.01	5.2 ± 1.6	100.0 ± 0
0.03	27.4 ± 3.2	96.7 ± 4.7
0.04	36.2 ± 6.2	94.2 ± 3.4
0.05	40.9 ± 2.8	76.1 ± 3.5
none (control)	—	95.8 ± 0.5

^a^Mean ± SD., *n* = 3.

In the case of pre-BACs, treatment at 0.05 w/v% of R-CFPT achieved probe uptake in nearly all of the cells (95.0 ± 4.7%) (Fig. 3b). We did not observe any dead pre-BACs in the PI assay under this condition. Due to the lack of cytotoxicity, the high incorporation efficiency and sufficient fluorescence intensity of intracellular R-CFPT under fluorescence microscopy observation at this high concentration of R-CFPT (0.05 w/v%), further optimization was not performed.

### Intracellular temperature change induced by uncoupler stimulation in BACs

**Figure 4:**
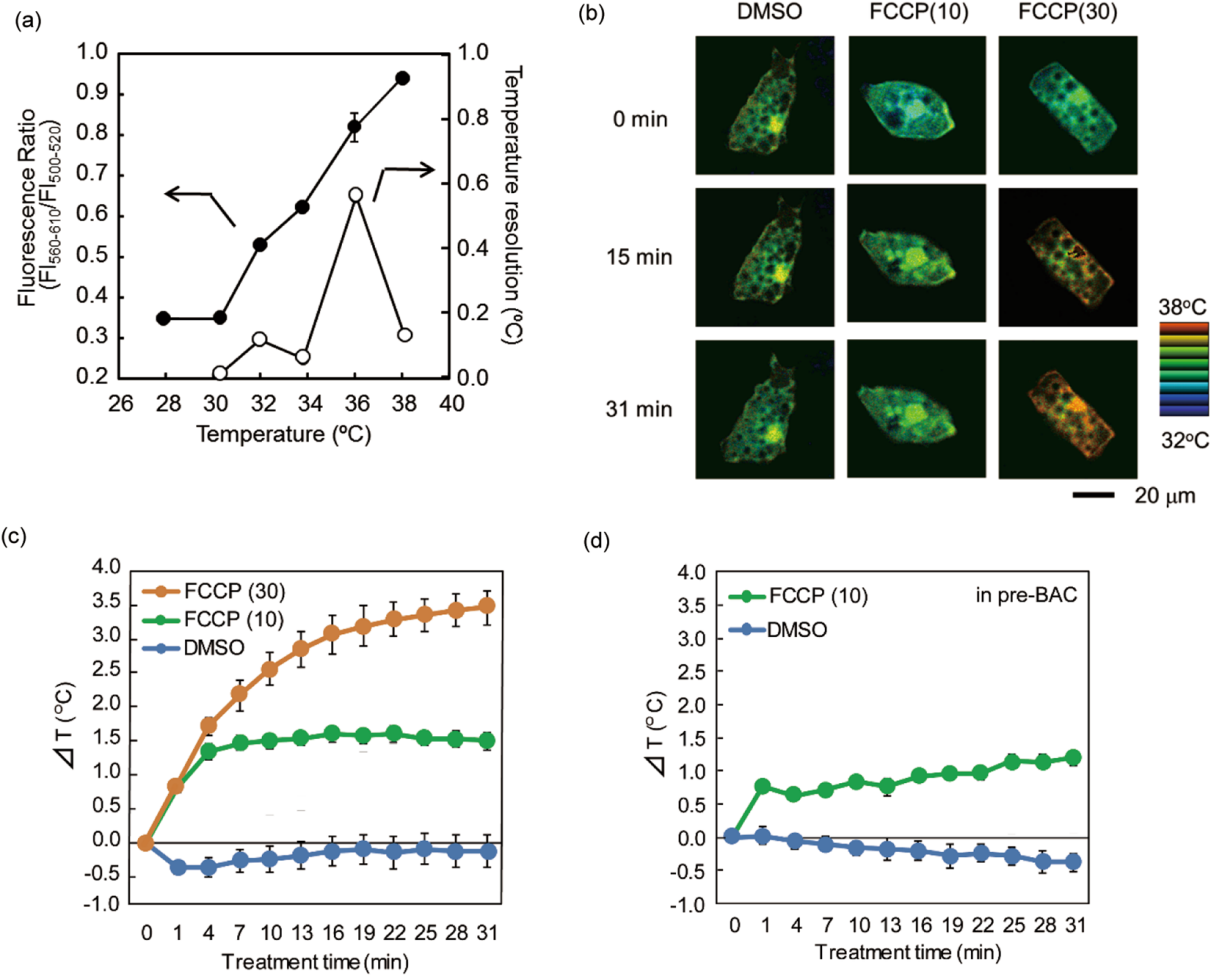
Intracellular temperature imaging analysis in BACs and pre-BACs in response to uncoupler stimuli using intracellular thermometer R-CFPT. (**a**) Fluorescence response (closed, left axis, n = 3, mean ± SD) and temperature resolution (open, right axis) of R-CFPT in a BAC extract under microscopy observation. FI_560–610_ and FI_500–520_ indicate the total fluorescence intensity between 560 and 610 nm and between 500 and 520 nm, respectively. Vertical bars indicate the SD based on three independent experiments. (**b**) Representative pseudocolor confocal images of the fluorescence ratio (Em. 560–610 nm/Em. 500–520 nm) in R-CFPT-incorporated matured BACs at 15 min and 31 min after 10 μM or 30 μM FCCP treatment. The control treatment was 0.1% DMSO. The ratio image is reconstructed in intensity-modulated display (IMD) mode. Bar, 20 µm. The temperature value in the pseudocolor bar was calculated from the temperature response curve shown in (**a**). (**c**) Intracellular temperature change during stimulation of matured BACs with FCCP (n = 8, 9 or 9 cells for 0.1% DMSO, 10 μM FCCP or 30 μM FCCP, respectively; mean ± SE). (**d**) Intracellular temperature change during stimulation of pre-BACs with FCCP (n = 7 or 16 cells for 0.1% DMSO or 10 μM FCCP, respectively; mean ± SE). In these experiments, the temperature of the medium was maintained at 30 °C.

We next used the R-CFPT probe with confocal microscopy observation to measure the intracellular temperature change in BACs. The ratio of the two fluorescence intensities of R-CFPT in a BAC lysate displayed a temperature-dependent increase (Fig. 4a). The temperature resolution, meaning the statistical minimum temperature difference that can be significantly discriminated, was 0.06 to 0.56 °C within the range 32 to 38 °C (Fig. 4a) when measured under a confocal microscope (see Methods). These results indicate that measured temperature changes can be discussed from the viewpoint of biological phenomena only if the temperature changes are larger than the temperature resolution.

To confirm the functionality of R-CFPT within a BAC, we measured the heat production induced by the mitochondrial uncoupler carbonyl cyanide *p*-trifluoromethoxyphenylhydrazone (FCCP)^43^, which is known to accelerate thermogenesis in mitochondria^18^. Representative intracellular temperature imaging after FCCP treatment calculated from the ratio of two simultaneously acquired fluorescence images derived from DBThD-AA (560–610 nm) and BODIPY-AA (500–520 nm) is shown in pseudocolor in Figs. 4b and S2 in Supplementary Information. Whereas the DMSO control did not induce changes in the fluorescence ratio, FCCP increased the temperature throughout cells, as shown by the higher fluorescence ratio. Furthermore, the intracellular temperature increase induced by 30 μM FCCP was higher than that induced by 10 μM FCCP in matured BACs. The average cellular temperature changes calculated from the time-lapse images of R-CFPT fluorescence in the BACs indicated that heat was generated immediately after stimulation by the uncoupler FCCP (Fig. 4c). As shown in Fig. 4d, 10 μM FCCP also stimulated heat production in pre-BACs, whereas 30 μM FCCP caused cell death, as indicated by obvious cell morphological changes (Fig. S3), such that temperature data were not available. As shown in Fig. 4c and d, the maximum temperature elevation caused by 10 μM FCCP was 1.60 ± 0.11 °C for BACs (at 22 min, n = 9 cells, mean ± SE) and 1.20 ± 0.11 °C for pre-BACs (at 31 min, n = 16 cells, mean ± SE). A significant difference in the maximum temperature increase between BACs and pre-BACs was confirmed by t-test (*p* = 0.0268). Furthermore, when the temperature of the medium was maintained at 30 °C, the intracellular temperature of pre-BACs and BACs was significantly different at 32.3 ± 0.2 °C (mean ± SE, n = 22) and 34.4 ± 0.2 °C (n = 50), as confirmed by Student’s t-test (*p* < 0.001). In contrast, the temperature of the culture medium during the experiments was monitored by R-CFPT and a thermocouple, and no changes in temperature were detected (Fig. S4). This indicates that the temperature increase in BACs was due to endogenous thermogenesis and not to global heating of the culture medium.

### Intracellular temperature change induced by β3-AR agonist stimulation in BACs

**Figure 5:**
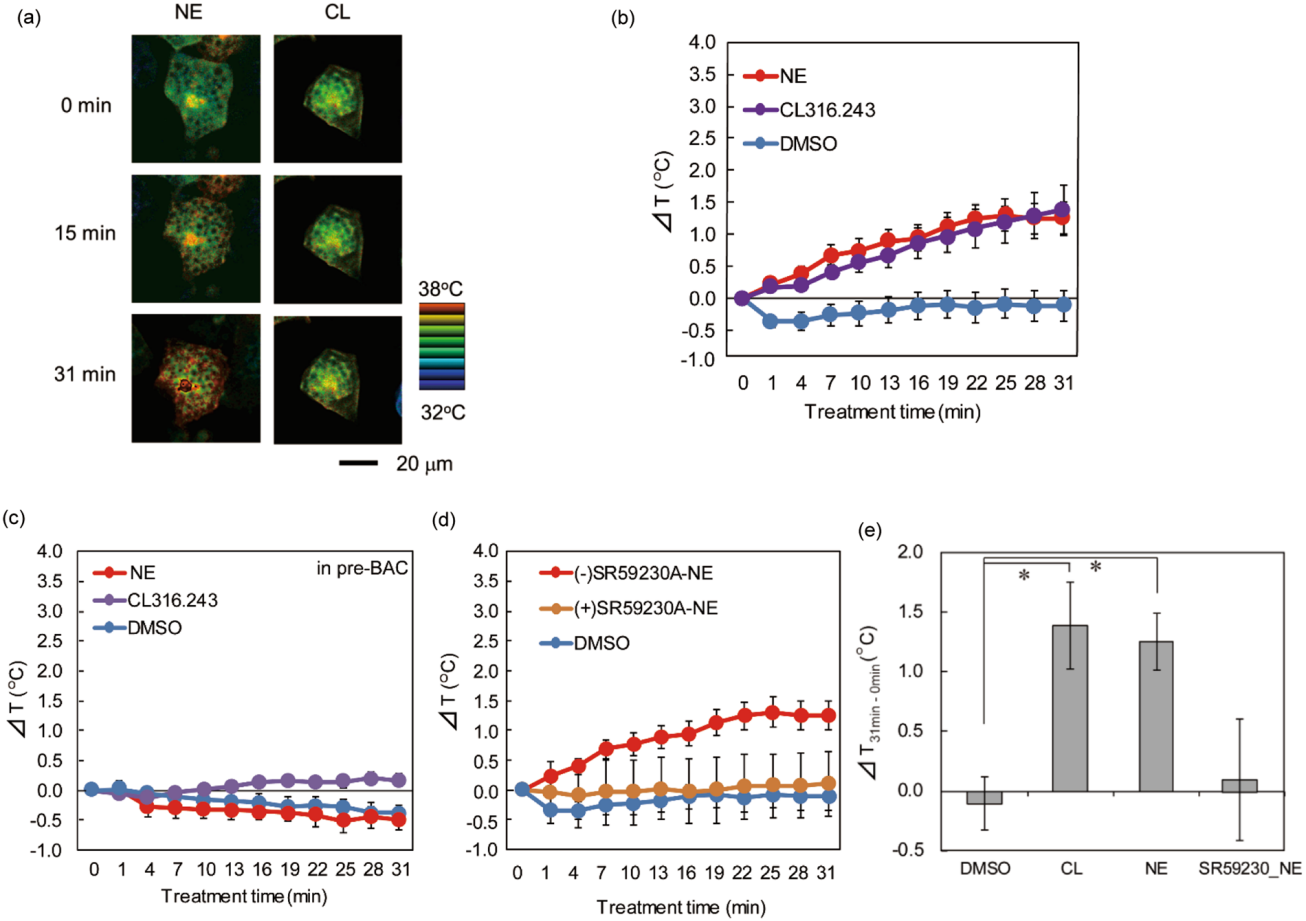
Intracellular temperature imaging analysis in BACs and pre-BACs in response to β-AR agonist stimuli using R-CFPT. (**a**) Representative pseudocolor confocal images of the ratio (Em. 560–610 nm/Em. 500–520 nm) in R-CFPT-incorporated matured BACs at 15 min and 31 min after 0.5 μM NE or 0.5 μM CL316.243 (CL) treatment. The ratio image is reconstructed in IMD mode. Bar, 20 µm. The temperature value in the pseudocolor bar was calculated from the temperature response curve shown in Fig. 4a. (**b**) Intracellular temperature change during stimulation of matured BACs with β-AR agonists (n = 8, 12 or 16 cells for 0.1% DMSO, 0.5 μM NE or 0.5 μM CL316.243, respectively; mean ± SE). (**c**) Intracellular temperature change during stimulation of pre-BACs with NE or CL316.243 (n = 7 or 22 cells for 1 μM NE or 1 μM CL316.243, respectively; mean ± SE). (**d**) Effect of pre-incubation (4 h) with 10 µM SR59230A on NE-induced temperature increase in matured BACs (n = 8, 12 or 14 cells for 0.1% DMSO, 0.5 μM NE without SR59230A or 0.5 μM NE with SR59230A, respectively; mean ± SE). In these experiments, the temperature of the medium was maintained at 30 °C. (**e**) Statistical analysis of NE-stimulated thermogenesis in BACs. The intracellular temperature change is shown during stimulation with β-AR agonists (n = 8, 12, 16 or 14 cells for 0.1% DMSO, 0.5 μM NE, 0.5 μM CL316.243 or 0.5 μM NE with SR59230A, respectively; mean ± SE). *P < 0.05 (n = 8–16) by one-way ANOVA followed by Dunnett’s test.

We next investigated whether stimulation with β-AR agonists would induce an intracellular temperature change in BACs. Representative intracellular temperature images after application of the β-AR agonists NE and CL316.243 are shown in pseudocolor in Figs 5a and S2. The results demonstrated that these β-AR agonists induced intracellular thermogenesis. As shown in Fig. 5b, the mean temperature increase at 31 min after NE and CL316.243 application was 1.25 ± 0.25 °C (mean ± S.E.) and 1.39 ± 0.38 °C, respectively. No significant difference in temperature change was observed between the two treatments. In contrast, no temperature change was observed in pre-BACs after β-AR agonist treatment (Fig. 5c), suggesting that intracellular thermogenesis induced by β-AR activation is specific to matured BACs.

To confirm the induction of thermogenesis by β3-AR activation, we evaluated the intracellular temperature of BACs pretreated with a specific β3-AR antagonist. Lipolysis assays indicated that SR59230A (10 μM) adequately suppressed NE-evoked lipolysis after 4 h of treatment, but not after 2 h of treatment (Fig. S5), which is consistent with the results of Tonello *et al*.^44^. The intracellular temperature of SR59230A-pretreated BACs remained almost constant despite NE stimulation (Fig. 5d). Statistical analysis also indicated that the cellular temperature change observed at 31 min after NE and CL316.243 treatment was significantly different from that after control treatment. In contrast, no significant differences in intracellular temperature change were observed between NE stimulation after pretreatment with SR59230A and the control (Fig. 5e). Together, these results strongly indicate that the thermogenesis induced by NE in BACs occurs through β3-AR activation. Finally, we evaluated whether there was a correlation between the cell temperature before stimulation and the degree of intracellular temperature change due to the stimulus (Fig. S6). No clear correlation was observed with any stimulus, including FCCP, NE and CL 316.243.

## Discussion

In this study, we have optimized a method to measure the intracellular temperature in primary cultured BACs by a CFPT for ratiometric sensing. The phenotype of primary cultured BACs is highly dependent on the culture conditions and origins of the precursor cells. For example, under non-optimized conditions, no UCP1 expression is detected even after NE stimulation^45^. The primary cultured BACs used in this study showed significant UCP1 protein expression (Fig. 2c), BAC-specific gene expression (Fig. 2b), and increased expression of UCP1 after NE stimulation (Figs. 2d and e). Optimization of the conditions for application of R-CFPT to BACs demonstrated an incorporation of R-CFPT into 36.2% of cells with low cytotoxicity. In previous studies, a non-ratiometric and cationic thermometer, termed FPT, was incorporated into approximately 50% of NIH3T3 cells and COS7 cells^20^, while R-CFPT was incorporated into almost all MOLT-4 and HEK293T cells^35^. Thus, introducing the polymeric thermometer into primary cultured BACs seems to be more challenging than its introduction into other cell lines.

The temperature resolution of R-CFPT evaluated in a BAC extract under microscopy—that is, 0.06 to 0.56 °C in the temperature range 32 to 38 °C (Fig. 4a)—seems to be sufficient for detecting the production of heat within BACs. The increases in cellular temperature were almost 1 °C or more during stimulation with FCCP or NE (Figs. 4c and 5b). The high temperature resolution of our method revealed differences in the intracellular temperature at steady state between matured BACs and pre-BACs.

To test the fluorescent polymeric thermometer, we examined whether uncoupling the mitochondrial membrane potential would lead to an increase in intracellular temperature. Plotting the time course of average cellular temperature changes calculated from the time-lapse images of R-CFPT in BACs indicated that heat generation was induced immediately after stimulation with the uncoupler FCCP (Fig. 4c). This result is comparable to that of a previous report in which an FCCP stimulus initiated a significant drop in mitochondrial inner membrane potential within 5 min of application, with a continued decrease in the potential for 30 min^46^. Moreover, in a recent study, treatment with the uncoupler CCCP, an analog of FCCP, was shown to increase neuronal cell temperature within 1 min of application^25^. Thus, our findings show that an uncoupler induces rapid heat generation in BACs comparable to its effect in other cell types.

Previous reports using several intracellular thermometers based on different detection systems have indicated that uncoupler treatment increases the intracellular temperature^18,21,25,27,28,34,47^. In this study, we demonstrated a difference between the intracellular temperature of matured BACs and that of pre-BACs. Furthermore, the increase in intracellular temperature at 31 min after application of 30 μM FCCP to BACs (mean 3.46 °C) was much higher than that reported previously for any other cell type or for pre-BACs in this study. This large temperature increase may be caused by the numerous mitochondria in matured BACs, as an uncoupler induces the release of heat from the mitochondria due to inhibition of ATP synthesis^48^. The concentration of mitochondrial protein is 20- to 30-fold higher in matured BACs than in pre-BACs^49^. One reason for the weak correlation between the mitochondrial mass and the increase in intracellular temperature may be the different expression of UCP1 protein in mitochondria in the two cell types; the mitochondria of the BACs used in this study dissipate heat modestly even in the unstimulated state due to the expressed UCP1 protein. In fact, the baseline cellular temperature of matured BACs was approximately 2 °C higher than that of pre-BACs. The influence of UCP1 on the intracellular temperature will be clarified in future studies using UCP1 knock-out or knock-down cells.

Next, we focused on the cellular temperature change induced by β3-AR stimulation. CL316.243, a selective β3-AR agonist, increased the intracellular temperature by the same degree as NE stimulation (Fig. 5b). Furthermore, although NE activates β1-, β3-, and α1-ARs, which are all expressed on the surface of BACs^50^, the β3-AR antagonist SR59230A inhibited the heat production stimulated by NE. Thus, our intracellular thermometry experiments demonstrated that β3-AR activation induces thermogenesis, which is consistent with the results of previous reports^51,52^. Further, the temperature increase induced by the β3-AR agonists occurred only in matured BACs, not in pre-BACs, demonstrating that thermogenesis in response to β3-AR agonist stimulation is specific to matured BACs.

Other recent studies have used various methods to measure changes in BAC temperature due to β3-AR activation^22,27,34,53^. Sato *et al*. used a temperature-responsive microcantilever to detect an increase of approximately 0.2 °C in cellular temperature during stimulation with 1 μM NE (>60 min)^53^, whereas the temperature increase in our study was 1.2 °C at 30 min after NE stimulation (Fig. 5b). As discussed by Sato *et al*.^53^, the temperature increase in their study was relatively small because their experiments were conducted at low temperature (25 ± 1 °C). In addition, there are likely other differences between our study and theirs, such as the physiological condition of the BACs derived from preparation. Consumption of O_2_ reaches a peak within 5 min of NE application to BACs^54^. However, the heat generation at 30 min was also relatively low in the experiment using a microcantilever^53^.

Kriszt *et al*. reported the microscopic visualization of thermogenesis in human brown adipocytes using a small organic fluorescent molecule accumulating in the ER^34^. The temperature of the ER in matured brown adipocytes was also elevated in response to β3-AR activation while precursor cells showed no changes in ER temperature. Hattori *et al*. reported that the intracellular temperature of BACs was elevated by approximately 1.3 °C in response to CL316.243 stimulation using hydrophilic fluorescent nanogel thermometers, which are localized in the cytoplasm^22^. This elevation is comparable to the intracellular temperature increase we observed using R-CFPT localized in the nucleus and the cytoplasm. These results suggest that the intracellular temperature change in response to CL316.243 occurred throughout the BACs. As Kiyonaka *et al*.^27^ reported, the temperature within BAC mitochondria is also elevated by stimulation with 10 μM NE. Although there is little description of the quantitative temperature rise in this report, the calibration curve suggests a mitochondrial temperature increase of a few degrees. As observed by transmission electron microscopy, the mitochondrial volume density of matured BACs is approximately 30% per cell volume^55^. In addition, in the present study, the intracellular temperature was calculated from the average of ratio intensity per entire single cell (see Methods). The NE-induced increase in intracellular temperature measured in this study is comparable to that measured by a fluorescent protein sensor^27^. The temperature rise of BACs by several degrees due to the β3-AR stimulation could be confirmed despite methodological differences (microcantilever^53^, small organic molecule^34^, polymer nanogel^22^ and a fluorescent protein^27^), suggesting that the intracellular temperature elevation is not an artifact.

Regarding the intracellular temperature distribution, our results showed that the temperature of nuclei (Figs. 4b and 5a) was higher than that of cytoplasm, which is consistent with the findings of previous reports in COS7 and HeLa cells with other polymeric thermometers and a thermometer consisting of two fluorescent proteins^18,20,28^. Previous reports showed that the temperature in the vicinity of mitochondria in COS7 cells and the temperature of the ER in brown adipocytes also increase for 30 min or longer after stimulation by FCCP and forskolin^18,34^. These findings are consistent with observations in the present study (Figs.  4b and 5a). We speculate that a constant thermodynamic steady state has been maintained due to continuation of heat production from mitochondria.

Finally, we discuss the validation of our intracellular thermometry using R-CFPT. Our results showed that the intracellular temperature of BACs was elevated by ~3 °C by chemical stimulation (FCCP and NE). The temperature measurements outside the cells indicated that the intracellular temperature increase was due to endogenous thermogenesis and not from an external heat source (Fig. S4 in Supplementary Information). Baffou *et al*.  previously stated that a single biological cell does not have sufficient energy for a temperature elevation of 1 °C based on the conventional law (Equation 1):

1 $${\rm{\Delta }}{\rm{T}}=\frac{P}{\kappa L}$$

where P is the heat power, κ is the thermal conductivity of cells, and L is the diameter of the heat source^56^. The reason for the discrepancy between experimental data and the theoretical value derived from Eq. 1 has often been discussed in the field of intracellular thermometry. In fact, the temperature increase in a single live cell has been measured in various kinds of cell lines using thermometers with different functional mechanisms: a few degrees in mitochondria in BACs by GFP^27^; 0.2 °C in BACs by a microcantilever^53^; 0.6 °C in U251 cells by a thermocouple^57^; 1.8 °C in NIH/3T3 cells by a quantum dot^24^; and 1.7 °C in ER in HeLa cells by an organic fluorescent molecule^33^. The commonality in the intracellular temperature increase strongly suggests that the observed temperature increases in cells are not artifacts. Furthermore, Baffou *et al*. stated that cellular temperature changes measured by fluorescence-based thermometry reflect factors other than the temperature, such as pH and ion strength. However, no alteration in the cell size of brown adipose cells that could cause a rapid change of intracellular ion-strength was observed during both uncoupler and β3-AR stimulation (Figs. 4b and 5a). The previous report also showed that the stimulation using NE changed the intracellular pH in BACs by only 0.2^58^. Considering the independence of the fluorescence properties of R-CFPT from pH and ionic strength^35^, this suggests that the temperature change in BACs observed in this study does not reflect changes in intracellular pH and ionic strength. Careful evaluation of each term in Eq. 1 might satisfy both experimental and theoretical researchers. Baffou *et al*.  used 0.1 nW for the heat source of a single cell, but it has been reported that BACs produce much more heat (~1.6 nW) per cell^59,60^. In addition, the thermal conductivity of a biological cell is estimated to be larger than that of water, which was used in Eq. 1 in the original discussion. Further experimental data on cellular thermodynamics will be helpful in establishing that a single cell can substantially raise its temperature by endogenous thermogenesis.

In summary, here we have developed a method to measure temperature within primary cultured BACs using a CFPT. The significant difference in responsiveness to the stimulation of an uncoupler and β-AR agonists between undifferentiated cells and mature cells was observed by analyzing average temperature changes of whole cells. Stimulation with a β3-AR agonist increased the temperature of matured BACs by approximately 1 °C, but did not affect immature pre-BACs. Furthermore, pretreating mature BACs with a β3-AR antagonist completely inhibited the increase in intracellular temperature. R-CFPT has a functional advantage in that incorporation of R-CFPT into multiple living cells can be achieved without special devices. Therefore, it will be relatively easy to apply it to high-throughput screening of candidate materials to increase BAC thermogenesis. This method will be applicable to studies of the mechanism of thermogenesis of BACs and screening for a material that can elevate thermogenesis in the fight against obesity.

## Methods

### Materials

Dulbecco’s modified eagle’s medium (DMEM) was obtained from Life Technologies (Carlsbad, CA, USA). Dexamethasone (DEX), 3-isobutyl-1-methyl-xanthine (IBMX) and insulin (bovine pancreas) were purchased from Sigma (St. Louis, MO, USA).

### BAC primary cell culture

Animal handling and primary cell culture was conducted in accordance with the guidelines for ethical animal care, handling, and termination of Kirin Company, which are in line with international and Japanese guidelines for animal care and welfare. This study was approved by the Kirin animal experiments committee. Three-week-old male Wistar rats were purchased from Charles River Japan (Tokyo, Japan). Isolation and culture of pre-BACs were performed as described^61^ with modification. Briefly, a rat was sacrificed, and interscapular depots of brown fat were resected and cut into small pieces. The tissue fragments were shaken in Krebs-Ringer bicarbonate HEPES buffer containing 3 mg/ml of collagenase for 30 min at 37 °C. The digested tissue was filtered through a nylon screen with 100-μm pore size. The filtrate was centrifuged at 400 *g* for 5 min at room temperature. The pellets, which consisted of the stromal-vascular fraction (SVF) of the tissue, were suspended in “growth medium”, comprising DMEM (4.5 g/L glucose, L-glutamine, sodium pyruvate) supplemented with 10% fetal calf serum (FCS), 17 μM D-pantothenic acid, 33 μM d-biotin, 100 μM ascorbic acid, 1 μM octanoic acid, 100 units/ml of penicillin, 0.1 mg/ml of streptomycin and 50 nM 3,3′,5-triiodo-L-thyronine (T3), and plated on 100-mm petri dishes. After incubation (5% CO_2_, 37 °C) for 3–4 days to give confluent pre-adipocytes (designated as “day 0”), differentiation was induced by replacing with fresh growth medium supplemented with 0.5 mM IBMX, 2.5 μM DEX, and 10 ng/ml of insulin. At day 2, the medium was changed to growth medium without FCS and supplemented with medium containing 0.1 nM insulin (maturation medium) to mature cells. Matured BACs were obtained on day 5 of incubation (5% CO_2_, 37 °C).

### Quantitative real-time PCR

Total RNA was extracted from primary cultured BAC tissues with TRIzol reagent (Life Technologies) and purified using RNeasy (Qiagen, Hilden, Germany) in accordance with the manufacturers’ instructions. cDNA was synthesized from total RNA by reverse transcription using the PrimeScript™ RT Master Mix (Takara Bio, Shiga, Japan). Quantitative real-time PCR was performed with a LightCycler 480 instrument (Roche Diagnostics, Indianapolis, IN, USA) using SYBR Premix Ex Taq II (Takara Bio). Levels of mRNA were normalized to those of 18S rRNA. The sense and antisense primers are listed in Table 2.

**Table 2:** Primers used for real-time PCR analysis.

Gene	Forward (5′ to 3′)	Reverse (5′ to 3′)
CPT1β	AGCTGCTGCTTTCCTATCATGGGT	TGCGGAAATAGGCTTCGTCATCCA
FABP4	GGACTTGGTCGTCATCCGGTC	CGTAAACTCTTGTAGAAGTCACGCC
PGC-1α	CACAACCGCAGTCGCAACATGCTC	GGCTTCAGCTTTGGCGAAGCCTTG
PPARα	GCTCACGGAATTTGCCAAGGCTAT	TGATGTCGCAGAATGGCTTCCTCA
PPARγ	AGCATCAGGCTTCCACTATGGAGT	ATTCGCCCAAACCTGATGGCATTG
UCP1	CGAGCCAAGATGGTGAGTTCGACA	GTGGTGATGGTCCCTAAGACACCT
18S rRNA	TGGGGTTCAGCCACCGAGATTGA	TTGCAATCCCCGATCCCCATCAC

### Western blot analysis

Cell lysates were prepared in RIPA buffer (150 mM NaCl, 1% Nonidet P-40, 1% sodium deoxycholate, 0.1% sodium dodecyl sulfate, 25 mM Tris/HCl, pH 7.6) supplemented with Halt protease inhibitor cocktail (Thermo Fisher Scientific, MA, USA) and 1 mM phenylmethylsulfonyl fluoride (PMSF). Protein content was measured with a BCA protein assay kit (Thermo Fisher Scientific). Protein lysates were separated by SDS-PAGE and transferred to nitrocellulose membranes. Anti-UCP1 (32 kDa) rabbit polyclonal antibody (1:1000, catalog number 662045, Calbiochem, Darmstadt, Germany) and anti-α-tubulin (52 kDa) rabbit polyclonal antibody (1:1000, catalog number 2125, Cell Signaling Technology, Danvers, MA, USA) were used as primary antibodies, and anti-rabbit IgG, HRP-linked whole antibody (1:5000, catalog number NA934-1ML, GE Healthcare Life Science, Little Chalfont, UK) was used as a secondary antibody. Antibody reactivity was detected using ECL Prime Western Blotting Detection Reagent (GE Healthcare Life Science). Band densities were evaluated by ImageJ software (NIH, USA).

### Fluorescent polymeric thermometer

The R-CFPT used in this study was prepared as previously described^35^.

### Introduction of R-CFPT into pre-BACs and BACs

R-CFPT was incorporated into pre-BACs and BACs without cell detachment. SVF samples isolated from rat interscapular BAT were cultured on a collagen-coated 35-mm glass-bottom dish (Matsunami Glass Ind., Osaka, Japan) in growth medium. The medium was removed, and the cells were washed with 5% glucose solution and then treated with 5% glucose solution containing either 0.05 w/v% (for pre-BACs) or 0.04 w/v% R-CFPT (for BACs). After incubation at 25 °C for 10 min, the cells were gently washed twice with HBSS. FluoroBrite™ DMEM (Life Technologies) supplemented with 15 mM HEPES was used for live-cell imaging. To measure the temperature of the culture medium, FluoroBrite DMEM supplemented with 15 mM HEPES containing 0.05 w/v% of R-CFPT was used.

### Confocal laser scanning imaging

Brown adipocytes treated with R-CFPT were observed by confocal laser microscopy (FV1000, Olympus, Tokyo, Japan) using a UPlanSApo 40 × lens (Olympus, N.A. 0.95) or a UPlanSApo 60 × lens (Olympus, N.A. 1.35). To evaluate the efficiency of R-CFPT incorporation into a BAC, the thermometer was excited at 473 nm and the emission between 485 and 585 nm was monitored. To measure the temperature by ratiometric sensing, emission at 500–520 nm and 560–610 nm was monitored. The temperature of the culture medium was controlled using a microscope cage incubation chamber (Water Jacket Top Stage Incubator H101, Oko-Lab, Pozzuoli, Italy). In the Oko-lab H101 system, the temperature of the medium was monitored every minute with a type K thermocouple placed approximately 5 mm away from the microscopic field and was controlled by a feedback mode with a temperature accuracy of ±0.1 °C (Fig. S7 in Supplementary information).

The chemical stimulus was applied by quickly adding 1 ml of pre-warmed chemical material in aqueous solution (culture medium containing 0.3% DMSO) to cells in 2 ml of culture medium. The temperature of the culture medium was maintained at 30 °C during analysis to perform the experiment within the linear response range of the calibration curve (as shown in Fig. 4a) and keep the temperature of the medium stable by reducing the deviation between room temperature and medium temperature.

The incorporation efficiency (%) was determined using Equation 2:

2 $$\begin{array}{c}{\rm{Incorporation}}\,{\rm{efficiency}}( \% )={\rm{number}}\,{\rm{of}}\,{\rm{cells}}\,{\rm{containing}}\,\\ \,\quad {\rm{R}}\mbox{-}\mathrm{CFPT}/{\rm{number}}\,{\rm{of}}\,{\rm{cells}}\times 100\end{array}$$

BACs in DMEM were treated with 0.01 mg/ml of Hoechst33342^62^, 1 μg/ml of NileRed^63^, and 1 μM MitoTrackerDeepRed FM (Life Technologies) (final concentration) at 37 °C for 15 min to stain nuclei, lipid droplet, and mitochondria, respectively. After staining, R-CFPT was incorporated into cells. Hoechst33342 was excited with a 405-nm laser, and emission between 410 and 510 nm was monitored. NileRed was excited at 559 nm, and emission between 570 and 625 nm was monitored. MitoTrackerDeepRed FM was excited at 635 nm, and emission between 650 and 750 nm was monitored. For observation of R-CFPT, excitation was carried out at 473 nm and emission was monitored between 480 and 510 nm.

### Imaging analysis

Images were analyzed with Fluoview (Olympus) and MetaMorph (Molecular Devices, USA). Intracellular mapping as an intensity-modulated display (IMD) was performed with MetaMorph. A ratio image was created by dividing each pixel of two images after processing with a median filter and background subtraction. The intracellular temperature was calculated from the average value of the ratio image of a whole single cell based on the calibration curve. To show the difference between precursor cells and mature BACs more accurately and more simply, we analyzed the intracellular temperature change using the average signal of the whole cell. Analysis for intracellular temperature change was performed after excluding the cells in which the fluorescence intensity became half or less after the stimulation.

### Measurement of the fluorescence response of R-CFPT in BAC extract

The fluorescence response of R-CFPT was evaluated in a BAC extract (0.01 w/v%) by confocal laser microscopy. The BAC extract was prepared as previously described^18^.

The temperature resolution (δT) of R-CFPT was evaluated using Equation 3 ^64^:

3 $$\delta T=(\frac{\partial T}{\partial {F}_{ratio}})\delta {F}_{ratio}$$

where ∂T/∂F_ratio_ and δF_ratio_ represent the inverse of the slope of the fluorescence ratio-temperature diagram and the SD of the fluorescence ratio, respectively. The SD was obtained by triplicate measurements of one sample at each temperature.

### Cell viability assay

A propidium iodide (PI) assay was used to determine cell viability, as described previously^35^. In brief, 0.5 mL of PI solution (2 μg/mL in PBS) was added to cells with introduced R-CFPT in 1 mL of FluoroBrite DMEM supplemented with 15 mM HEPES buffer, and cells were cultured at 37 °C for 30 min. Fluorescence images of PI were acquired by exciting the cells at 559 nm and recording the emission between 655 and 755 nm using the variable bandpass filter sets of a DM405/473/559 excitation dichroic mirror. Cell viability (%) was determined using Equation 4:

4 $$\begin{array}{c}{\rm{Cell}}\,{\rm{viability}}\,( \% )={\rm{number}}\,{\rm{of}}\,{\rm{PI}}\mbox{-}\mathrm{negative}\,{\rm{cells}}/{\rm{number}}\\ \phantom{\rule{7em}{0ex}}\quad {\rm{of}}\,{\rm{cells}}\,{\rm{containing}}\,{\rm{R}}\mbox{-}\mathrm{CFPT}\times 100\end{array}$$

### Lipolysis assay

Matured BACs were washed with FluoroBrite DMEM medium and then stimulated with 0.5 μM NE for 1 h. Cell-free supernatants were harvested, and glycerol content was determined using the free glycerol assay kit (Cayman Chemical Company, Ann Arbor, MI, USA) following the manufacturer’s protocol.

### Statistical analysis

Statistical differences were analyzed by appropriate statistical methods as specified in the figure legends. A *P* value of less than 0.05 was considered statistically significant. Statistical analysis was performed using EZR (Saitama Medical Center, Jichi Medical University, Saitama, Japan), which is a graphical user interface for R (The R Foundation for Statistical Computing, Vienna, Austria)^65^.

## Electronic supplementary material

Supplementary Figures and Table

## References

[1] Lowell BB, Spiegelman BM (2000). Towards a molecular understanding of adaptive thermogenesis. Nature.

[2] Cannon B, Nedergaard J (2004). Brown adipose tissue: function and physiological significance. Physiol.  Rev..

[3] Power GG (1989). Umbilical cord occlusion but not increased plasma T3 or norepinephrine stimulate brown adipose tissue thermogenesis in the fetal sheep. J.  Dev.  Physiol..

[4] Kajimura S (2009). Initiation of myoblast to brown fat switch by a PRDM16-C/EBP-beta transcriptional complex. Nature.

[5] Ahfeldt T (2012). Programming human pluripotent stem cells into white and brown adipocytes. Nat.  Cell Biol..

[6] Nishio M (2012). Production of functional classical brown adipocytes from human pluripotent stem cells using specific hemopoietin cocktail without gene transfer. Cell Metab..

[7] Yoneshiro T (2012). Nonpungent capsaicin analogs (capsinoids) increase energy expenditure through the activation of brown adipose tissue in humans. Am.  J.  Clin.  Nutr..

[8] Kim M (2015). Fish oil intake induces UCP1 upregulation in brown and white adipose tissue via the sympathetic nervous system. Sci.  Rep..

[9] Matsushita M (2015). Kaempferia parviflora extract increases whole-body energy expenditure in humans: roles of brown adipose tissue. J.  Nutr.  Sci.  Vitaminol..

[10] Morimoto-Kobayashi Y (2015). Matured Hop Bittering Components Induce Thermogenesis in Brown Adipose Tissue via Sympathetic Nerve Activity. PLoS One.

[11] Ricquier D, Bouillaud F (2000). The uncoupling protein homologues: UCP1, UCP2, UCP3, StUCP and AtUCP. Biochem.  J..

[12] Harms M, Seale P (2013). Brown and beige fat: development, function and therapeutic potential. Nat.  Med..

[13] Shinoda K (2015). Genetic and functional characterization of clonally derived adult human brown adipocytes. Nat.  Med..

[14] Klingenspor M (2003). Cold-induced recruitment of brown adipose tissue thermogenesis. Exp.  Physiol..

[15] Tharp KM (2015). Matrix assisted transplantation of functional beige adipose tissue. Diabetes.

[16] Nedergaard J, Cannon B, Lindberg O (1977). Microcalorimetry of isolated mammalian cells. Nature.

[17] Ricquier D, Gaillard JL, Turc JM (1979). Microcalorimetry of isolated mitochondria from brown adipose tissue. Effect of guanosine-di-phosphate. FEBS Lett.

[18] Okabe K (2012). Intracellular temperature mapping with a fluorescent polymeric thermometer and fluorescence lifetime imaging microscopy. Nat.  Commun..

[19] Tsuji T, Yoshida S, Yoshida A, Uchiyama S (2013). Cationic fluorescent polymeric thermometers with the ability to enter yeast and mammalian cells for practical intracellular temperature measurements. Anal.  Chem..

[20] Hayashi T, Fukuda N, Uchiyama S, Inada N (2015). A cell-permeable fluorescent polymeric thermometer for intracellular temperature mapping in mammalian cell lines. PLoS One.

[21] Gota C (2009). Hydrophilic fluorescent nanogel thermometer for intracellular thermometry. J.  Am.  Chem.  Soc..

[22] Hattori K (2016). ASK1 signalling regulates brown and beige adipocyte function. Nat.  Commun..

[23] Albers AE (2012). Dual-emitting quantum dot/quantum rod-based nanothermometers with enhanced response and sensitivity in live cells. J.  Am.  Chem.  Soc..

[24] Yang JM, Yang H, Lin L (2011). Quantum dot nano thermometers reveal heterogeneous local thermogenesis in living cells. ACS Nano.

[25] Tanimoto R (2016). Detection of Temperature Difference in Neuronal Cells. Sci.  Rep..

[26] Donner JS (2012). Mapping intracellular temperature using green fluorescent protein. Nano Lett..

[27] Kiyonaka S (2013). Genetically encoded fluorescent thermosensors visualize subcellular thermoregulation in living cells. Nat.  Methods.

[28] Nakano M (2017). Genetically encoded ratiometric fluorescent thermometer with wide range and rapid response. PLoS ONE.

[29] Takei Y (2014). A nanoparticle-based ratiometric and self-calibrated fluorescent thermometer for single living cells. ACS Nano.

[30] Ke G (2012). L-DNA molecular beacon: a safe, stable, and accurate intracellular nano-thermometer for temperature sensing in living cells. J.  Am.  Chem.  Soc..

[31] Shang L, Stockmar F, Azadfar N, Nienhaus GU (2013). Intracellular thermometry by using fluorescent gold nanoclusters. Angew.  Chem.  Int.  Ed.  Engl..

[32] Kucsko G (2013). Nanometre-scale thermometry in a living cell. Nature.

[33] Arai S (2014). A Molecular Fluorescent Probe for Targeted Visualization of Temperature at the Endoplasmic Reticulum. Sci.  Rep..

[34] Kriszt R (2017). Optical visualisation of thermogenesis in stimulated single-cell brown adipocytes. Sci.  Rep..

[35] Uchiyama S (2015). A cationic fluorescent polymeric thermometer for the ratiometric sensing of intracellular temperature. Analyst.

[36] Uchiyama S (2012). Environment-sensitive fluorophores with benzothiadiazole and benzoselenadiazole structures as candidate components of a fluorescent polymeric thermometer. Chem.  – Eur.  J..

[37] Gota C, Uchiyama S, Ohwada T (2007). Accurate fluorescent polymeric thermometers containing an ionic component. Analyst.

[38] Isidor MS (2016). An siRNA-based method for efficient silencing of gene expression in mature brown adipocytes. Adipocyte.

[39] Umekawa T, Yoshida T, Sakane N, Kondo M (1996). Effect of CL316,243, a highly specific beta 3-adrenoceptor agonist, on lipolysis of human and rat adipocytes. Horm.  Metab.  Res..

[40] Rosen ED, MacDougald OA (2006). Adipocyte differentiation from the inside out. Nat.  Rev.  Mol.  Cell Biol..

[41] Mercader J, Palou A, Bonet ML (2010). Induction of uncoupling protein-1 in mouse embryonic fibroblast-derived adipocytes by retinoic acid. Obesity.

[42] Krishan A (1975). Rapid flow cytofluorometric analysis of mammalian cell cycle by propidium iodide staining. J.  Cell Biol..

[43] Heytler PG, Prichard WW (1962). A new class of uncoupling agents–carbonyl cyanide phenylhydrazones. Biochem.  Biophys.  Res.  Commun..

[44] Tonello C (1998). SR59230A blocks beta3-adrenoceptor-linked modulation of upcoupling protein-1 and leptin in rat brown adipocytes. Eur.  J.  Pharmacol..

[45] Kajimoto K (2004). Identification of possible protein machinery involved in the thermogenic function of brown adipose tissue. J.  Med.  Invest..

[46] Mignen O (2005). Carboxyamidotriazole-induced inhibition of mitochondrial calcium import blocks capacitative calcium entry and cell proliferation in HEK-293 cells. J.  Cell Sci..

[47] Homma M (2015). A ratiometric fluorescent molecular probe for visualization of mitochondrial temperature in living cells. Chem.  Commun..

[48] Nakamura T, Matsuoka I (1978). Calorimetric studies of heat of respiration of mitochondria. J.  Biochem..

[49] Wilson-Fritch L (2003). Mitochondrial biogenesis and remodeling during adipogenesis and in response to the insulin sensitizer rosiglitazone. Mol.  Cell.  Biol..

[50] Lafontan M, Berlan M (1993). Fat cell adrenergic receptors and the control of white and brown fat cell function. J.  Lipid Res..

[51] Tanaka E (1995). Regulation of heat production of brown adipocytes via typical and atypical beta-adrenoceptors in the rat. Jpn.  J.  Physiol..

[52] de Souza CJ, Burkey BF (2001). Beta3-adrenoceptor agonists as anti-diabetic and anti-obesity drugs in humans. Curr.  Pharm.  Des..

[53] Sato MK (2014). Temperature changes in brown adipocytes detected with a bimaterial microcantilever. Biophys.  J..

[54] Prusiner SB, Cannon B, Lindberg O (1968). Oxidative metabolism in cells isolated from brown adipose tissue. 1. Catecholamine and fatty acid stimulation of respiration. Eur.  J.  Biochem..

[55] Uldry M (2006). Complementary action of the PGC-1 coactivators in mitochondrial biogenesis and brown fat differentiation. Cell Metab..

[56] Baffou G, Rigneault H, Marguet D, Jullien L (2014). A critique of methods for temperature imaging in single cells. Nat.  Methods.

[57] Wang C (2011). Determining intracellular temperature at single-cell level by a novel thermocouple method. Cell Res..

[58] Civelek VN (1996). Intracellular pH in adipocytes: Effects of free fatty acid diffusion across the plasma membrane, lipolytic agonists, and insulin. Proc.  Natl.  Acad.  Sci.  USA.

[59] Johannessen EA (2002). Micromachined Nanocalorimetric Sensor for Ultra-Low-Volume Cell-Based Assays. Anal.  Chem..

[60] Suzuki M (2015). The 10^5^ gap issue between calculation and measurement in single-cell thermometry. Nat.  Methods.

[61] Forest C (1987). Expression of the mitochondrial uncoupling protein in brown adipocytes. Absence in brown preadipocytes and BFC-1 cells. Modulation by isoproterenol in adipocytes. Exp.  Cell Res..

[62] Latt SA, Stetten G (1976). Spectral studies on 33258 Hoechst and related bisbenzimidazole dyes useful for fluorescent detection of deoxyribonucleic acid synthesis. J.  Histochem.  Cytochem..

[63] Greenspan P, Mayer EP, Fowler SD (1985). Nile red: a selective fluorescent stain for intracellular lipid droplets. J.  Cell Biol..

[64] Baker, S. N., McCleskey, T. M. & Baker, G. A. An Ionic Liquid-Based Optical Thermometer. in *Ionic Liquids IIIB: Fundamentals*, *Progress*, *Challenges*, *and Opportunities: Transformations and Processes*. (ed. Rogers, R. D. & Seddon, K. R.) 171–181 (ACS Symposium Series, 2005).

[65] Kanda Y (2013). Investigation of the freely available easy-to-use software ‘EZR’ for medical statistics. Bone Marrow Transplant.

