# Supplementary material for: Difference in intracellular temperature rise between matured and precursor brown adipocytes in response to uncoupler and β-adrenergic agonist stimuli
Source: Sci Rep. 2017 Oct 10;7:12889. doi: 10.1038/s41598-017-12634-7 (PMC5635136; doi:10.1038/s41598-017-12634-7)
Supplement: Supplementary file 1 — Supplementary Figures and Table [file 41598_2017_12634_MOESM1_ESM.pdf]

SUPPLEMENTARY INFORMATION

Difference in intracellular temperature rise between matured and precursor brown adipocytes in response to uncoupler and β-adrenergic agonist stimuli

Authors

Toshikazu Tsuji,*[a,b] Kumiko Ikado,[a] Hideki Koizumi,[a] Seiichi Uchiyama,[b] Kazuaki Kajimoto,[c]

[a]Central Laboratories for Key Technologies, KIRIN Company Limited, 1-13-5 Fukuura, Kanazawa-ku, Yokohama-shi, Kanagawa 236-0004, Japan. E-mail: Toshikazu_2_Tsuji@kirin.co.jp. Phone: +81-45-330-9005. Fax: +81-45-788-4042.
[b]Graduate School of Pharmaceutical Sciences, The University of Tokyo, 7-3-1 Hongo, Bunkyo-ku, Tokyo 113-0033, Japan. E-mail: seiichi@mol.f.u-tokyo.ac.jp. Phone/Fax: +81-3-5841-4768.
[c]Faculty of Pharmaceutical Sciences, Hokkaido University, Kita-12, Nishi-6, Kita-ku, Sapporo, Hokkaido 060–0812, Japan. E-mail: k-kajimo@pharm.hokudai.ac.jp. Phone: +81-11-706-3919. fax: +81-11-706-4879.

*Corresponding author

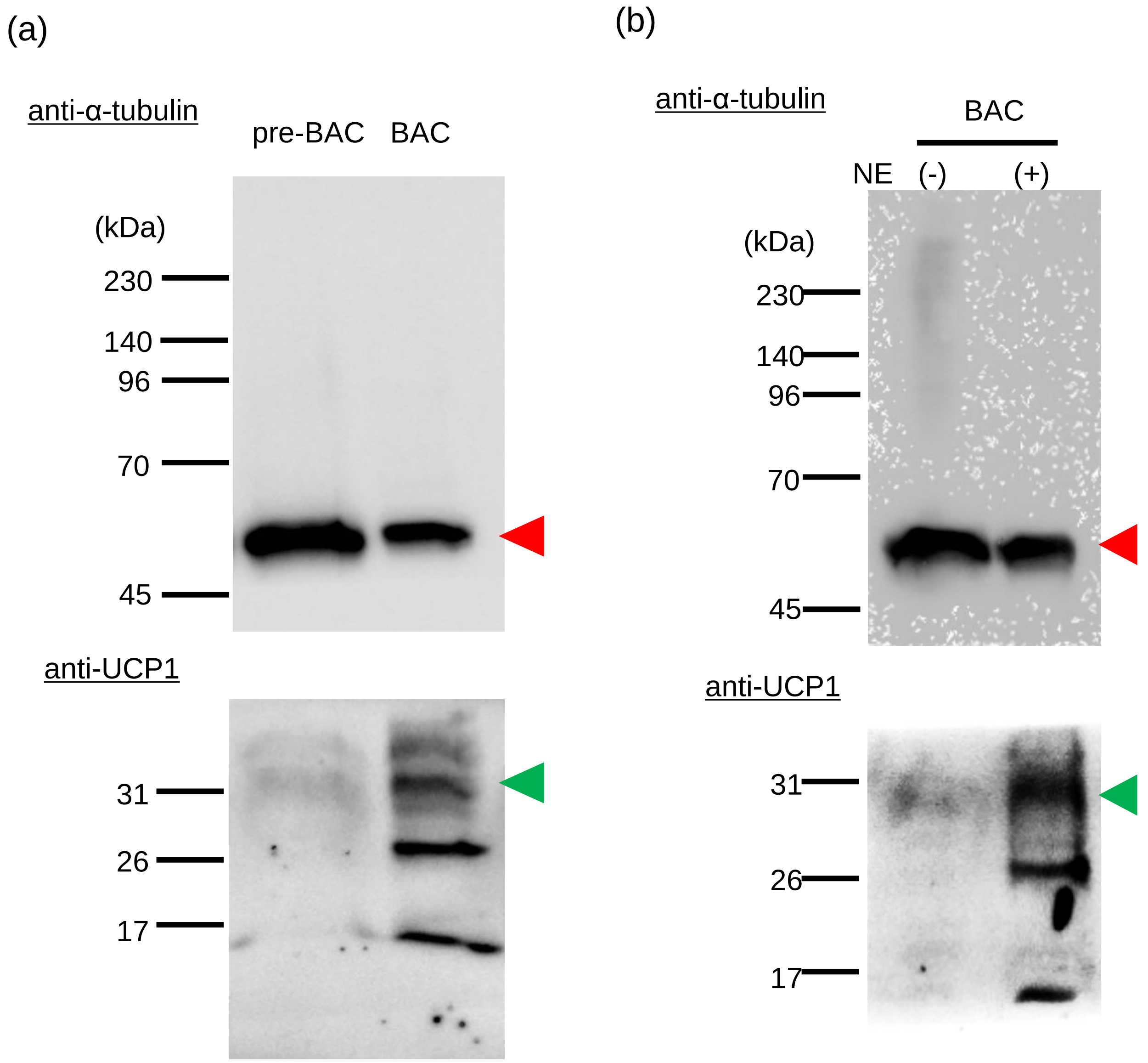

Figure S1 (a) Western blot analysis of UCP1 protein expression in pre-BACs and BACs with the full size panels for Figure 2c. (b) UCP1 protein expression in BACs in response to NE stimulation with the full size panels for Figure 2d. The red and green arrowheads indicate α-tubulin and UCP1, respectively.

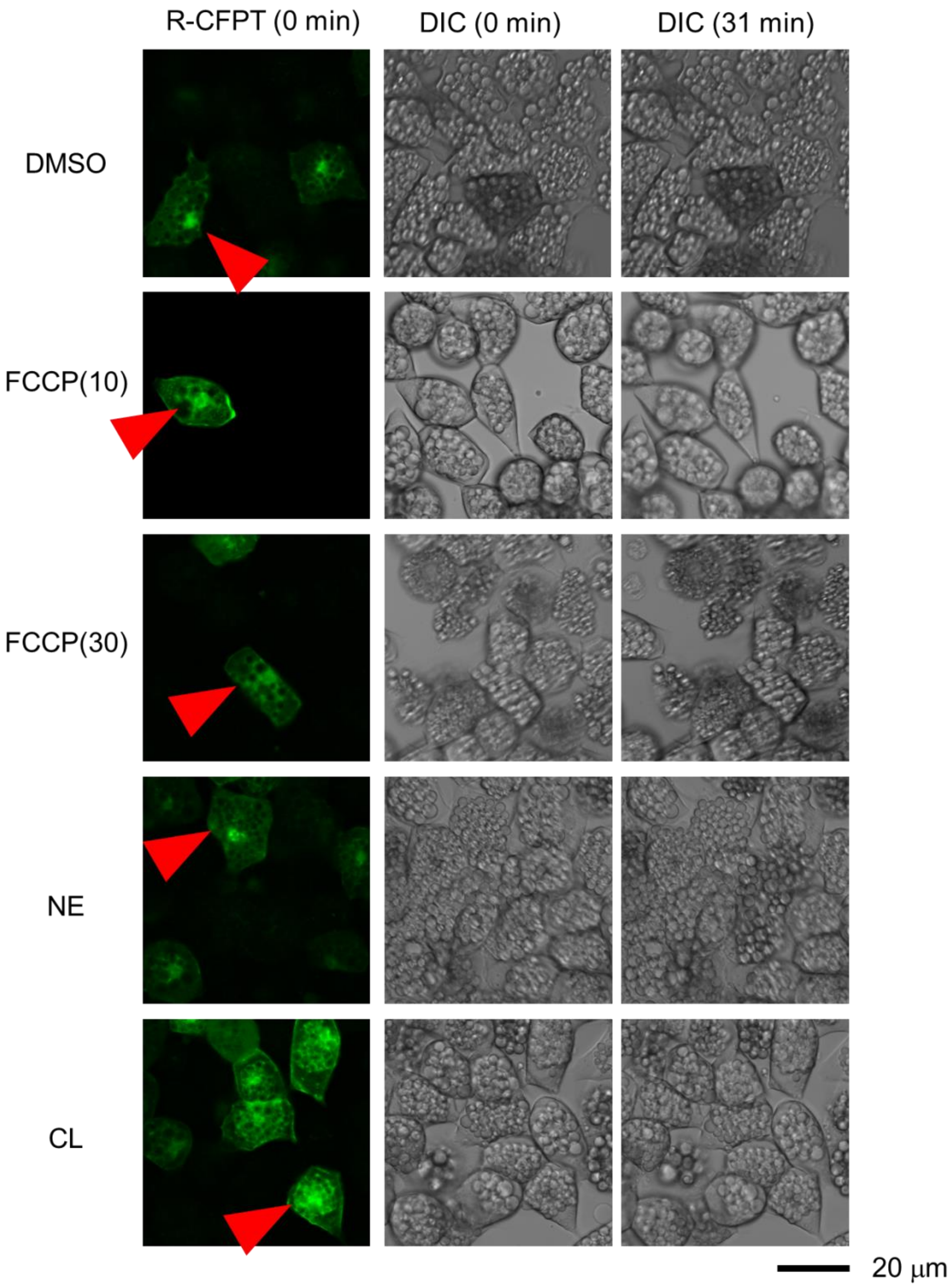

Figure S2 Wide field images of mature BACs before and after stimulation. The red arrowhead indicates the cell shown in Figures 4b and 5a. Bar = 20 μm.

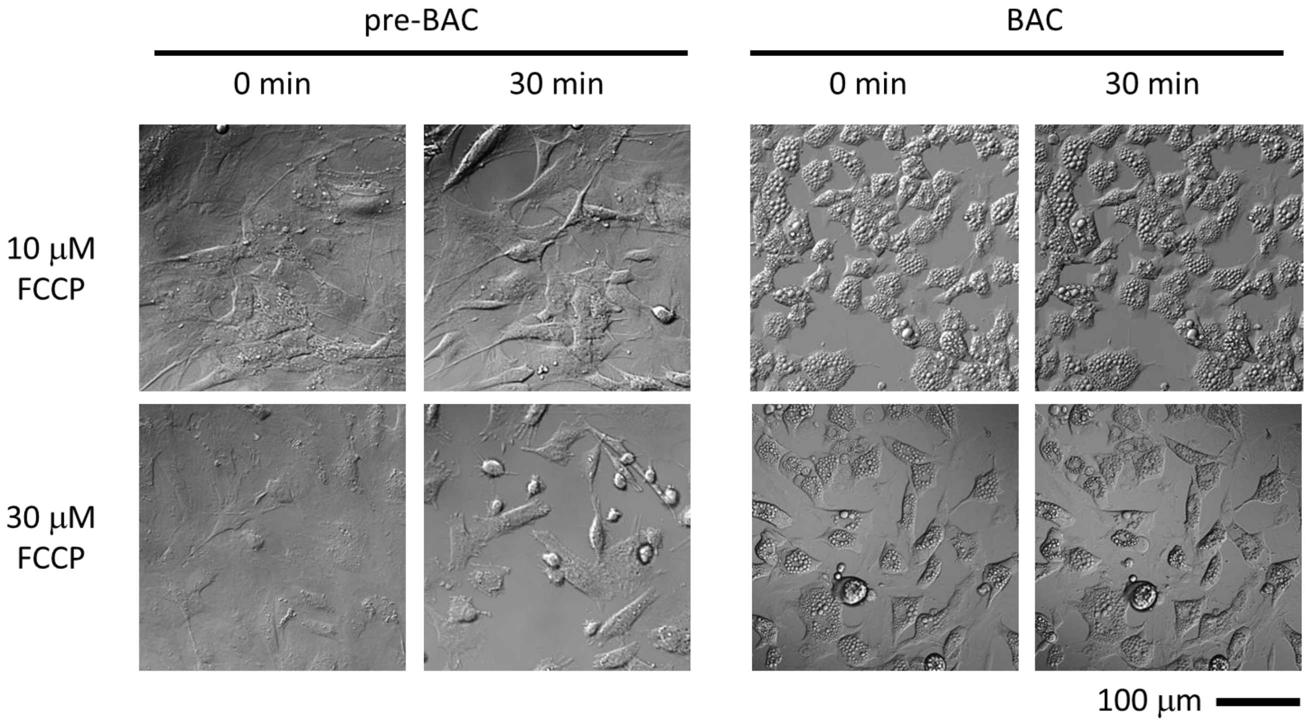

Figure S3 DIC images of pre-BACs and BACs after stimulation with FCCP. Bar = 100 μm.

Tsuji *et al.* Supplementary information in Figure S4

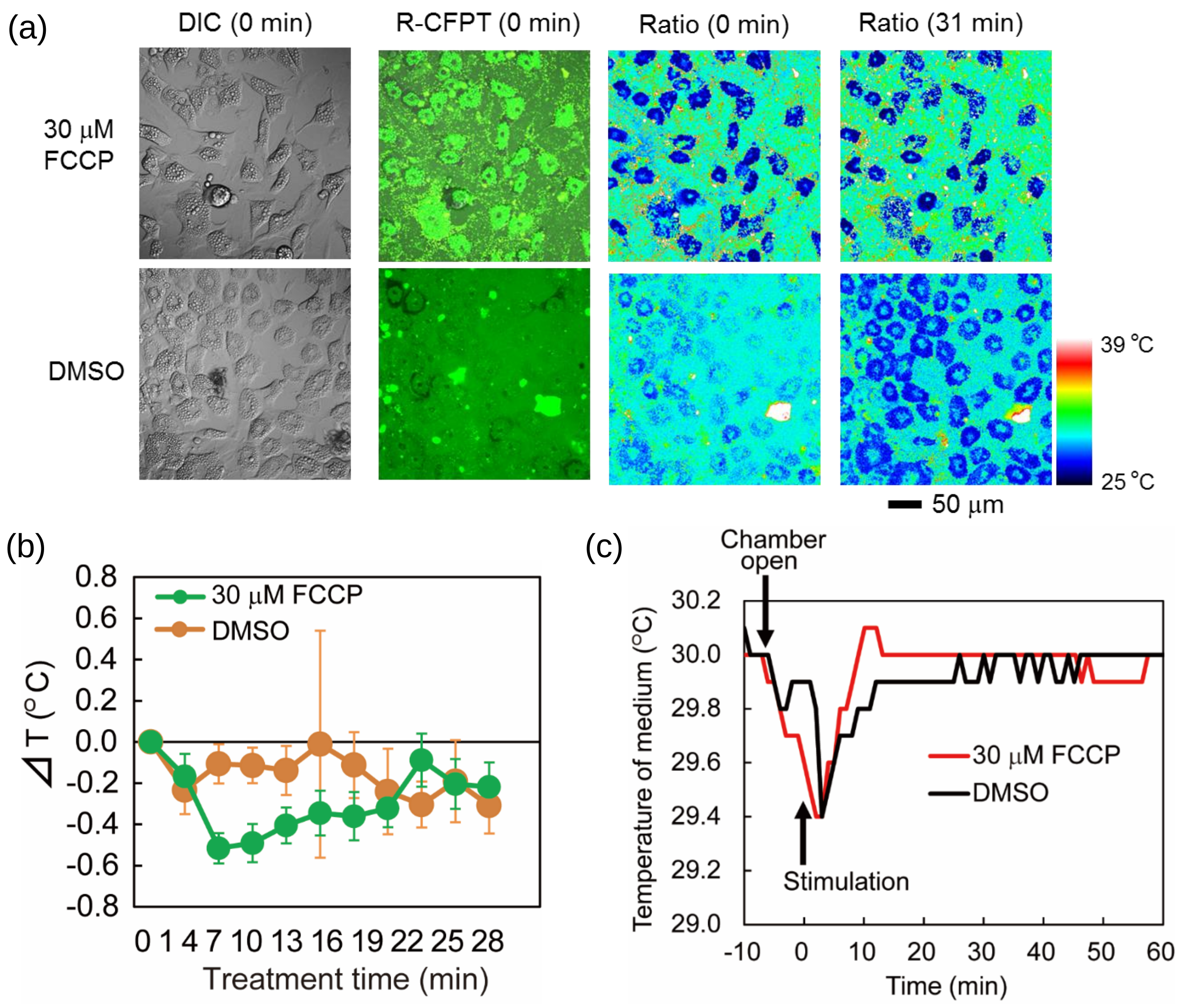

Figure S4 Temperature changes of the culture medium during the experiments of FCCP stimulation of BACs. (a) Representative DIC images (left), confocal fluorescence images of R-CFPT (centre left), pseudocolor confocal images of ratio (Em. 560-610 nm / Em. 500-520 nm) at 0 min (centre right) and pseudocolor images of ratio at 31 min (right) of BACs in culture medium (Fluorobrite DMEM with 15 mM HEPES) containing 0.05 w/v% R-CFPT at 30 °C. Temperature values in the pseudocolor bar were calculated from the temperature response curve shown in Fig. 4a. Bar = 50 µm. (b) Temperature changes of the culture medium during FCCP stimulation with R-CFPT (n = 19 or 10 ROIs in medium for 30 µM FCCP or 0.1% DMSO, respectively; mean ± SE). (c) Temperature changes of the culture medium during FCCP stimulation using a type K thermocouple placed approximately 5 mm away from the microscopic field. In these experiments, the temperature of the medium was maintained at 30 °C.

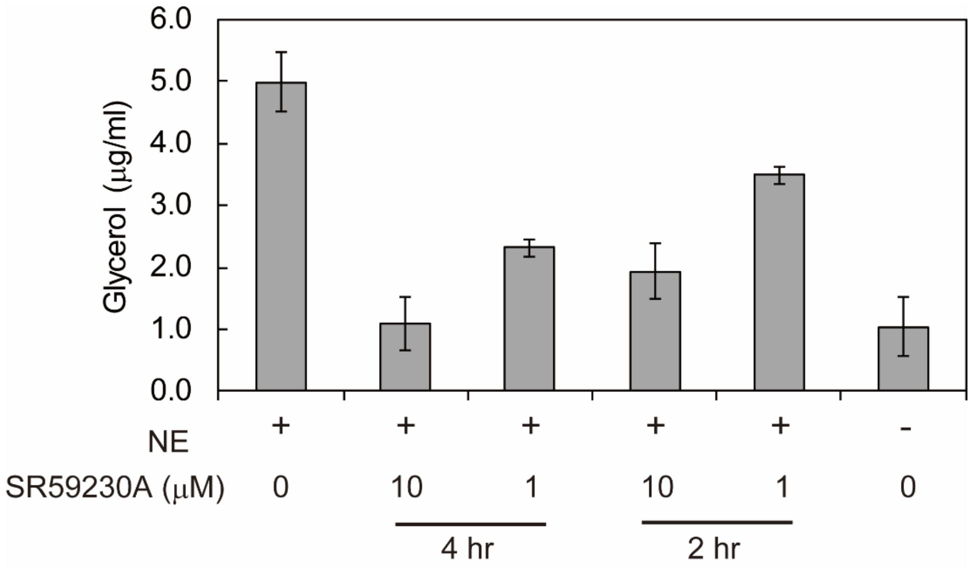

Figure S5 Inhibition of NE-stimulated lipolysis in BACs by the β3-AR antagonist SR59230A. BACs were incubated with or without the indicated amounts of SR59230A or control. The SR59230A stimulus was applied for 2 hr or 4 hr before NE stimulation. An aliquot of medium was collected, and the amount of glycerol released was measured as described in Materials and Methods. Results are expressed as the mean ± SE of triplicate measurements.

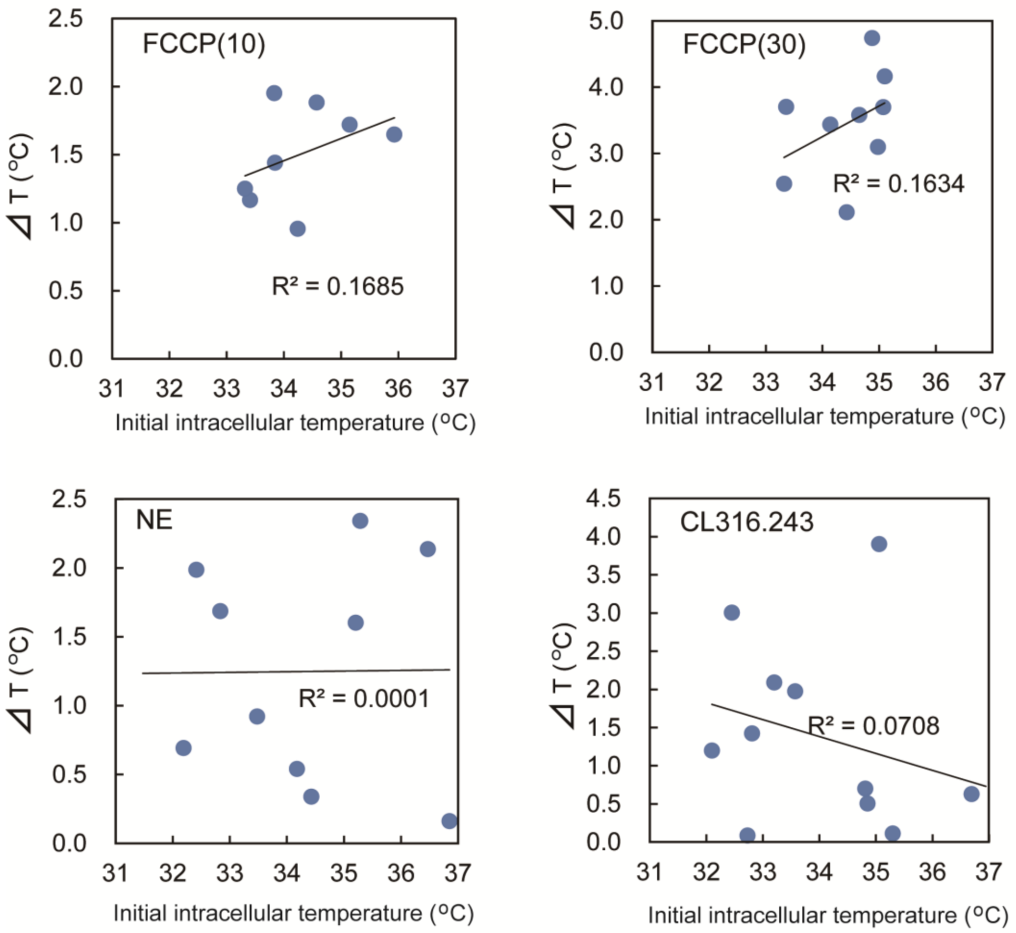

Figure S6 The relationship between initial intracellular temperature before stimulation and intracellular temperature change in response to chemical stimulation in a cell. ΔT indicates difference of intracellular temperature before and after treatment for 31 min with 10 μM FCCP (FCCP(10), 30 μM FCCP (FCCP(30), 0.5 μM NE (NE) or 0.5 μM CL316.243 (CL316.243). R^2 indicates the Pearson correlation coefficient between two variables: initial intracellular temperature and intracellular temperature change .

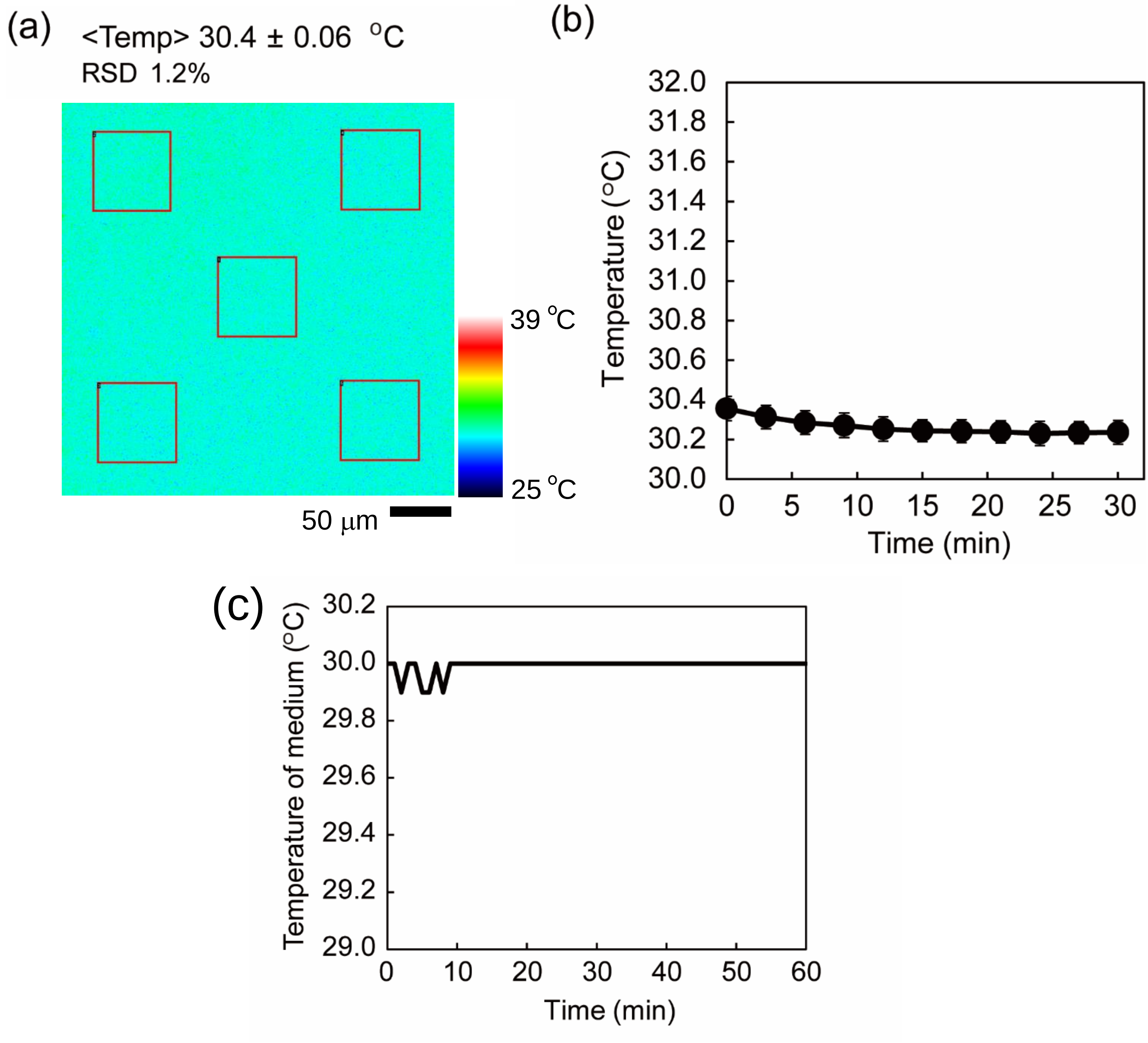

Figure S7 Internal temperature fluctuations in the microscopic field. (a) Pseudocolor confocal image of the ratio (Em. 560-610 nm / Em. 500-520 nm) in 0.01 w/v% R-CFP-containing medium (FluorobriteDMEM with 15 mM HEPES) at 30 ºC with x40 objective lens. Temperature fluctuations due to observed ROIs were evaluated. The average temperature and relative standard deviation (RSD) of five ROIs (red square) was calculated. Bar = 50 μm.

(b) Time course of temperature in the microcsopic field monitored by R-CFPT (0.01 w/v%). Shown is the mean and SD of the five ROIs in panel (a). (c) Time course of temperature in the microcsopic field monitored by a type K thermocouple placed approximately 5 mm away from the microcsopic field. In these experiments, the temperature of the medium was maintained at 30 ºC.

Table S1. Comparison of the properties of fluorescent thermometers for intracellular temperature measurement.

ref	Parameter [a]	Cell line	Temperature resolution (°C)	Temperature sensitivity (%/°C)	Distribution within cell	Introduction method	Acquisition time for an image
18	τ_f (500-700 nm)	COS7, HeLa	0.18–0.58	6.3 (35-40°C)	whole cell	microinjection	～1 min
19	τ_f (565 nm)	yeast, MOLT-4, HEK293T	0.09–0.78		cytoplasm (or whole cell)	20 min, 25°C	< 1 s
20	τ_f(500-700 nm)	HeLa, COS7, NIH/3T3	0.05–0.54		whole cell	10 min	～1 min
21	FI (515-550 nm)	COS7	0.29–0.50		dotted in cytoplasm	microinjection	< 0.5s
22	FI (515-550 nm)	mouse primary brown adipocyte	0.29–0.50		cytoplasm	microinjection	< 0.5s
23	FI ratio (630-640/664-674 nm)	HeLa, NIH/3T3	0.2			assisted by cationic polymer	
24	λ_{em}	NIH/3T3	(0.105 nm/°C)	0.016	dotted within a cell	1 h, 37°C, assisted by Qtracker	
25	FI ratio (650-670/630-650 nm)	SH-SY5Y	0.8-0.9	6.3	dotted in cytoplasm	1 h, 37°C	< 20 s
26	Fluorescence anisotropy	HeLa, U-87 MG	1.2		whole cell	genetically expressed	> 1 min
27	FI ratio (ex 400/480 nm)	HeLa, brown adipocyte, C2C12 myotube		3 (30-40°C)	cytoplasm, mitochondria, ER	genetically expressed	< 2 s
28	FI ratio (509/425 nm)	HeLa, medaka embryo	0.1-0.4	2.6	cytoplasm, mitochondria, nucleus	genetically expressed	< 2 s
29	Em ratio	HaLa	1		endosome	2 h, 37°C	
30	FI (505-535 nm)	HeLa	0.2–0.7		nucleus	4 h, 37°C by liposome transfection	
31	τ_f (655-725 nm)	HeLa	0.3–0.5		dotted within a cell	2 h, 37°C	
32	FI with microwave excitation	WS1	0.044		dotted within a cell	assisted by nanowire	
33	FI ratio (590-690 nm)	NIH/3T3, HeLa, C2C12, Chang, BAT	0.4		endoplasmic reticulum	30 min, 37°C	< 2s
34	FI (555-655 nm)	WT-1 and human brown adipocyte		4.8 (35.7-42.8°C)	endoplasmic reticulum	30 min, 37°C	< 2 s
35	FI ratio (580/515 nm)	HEK293T, MOLT-4	0.01–0.25	5.3 (30-40°C)	whole cell	10 min	< 1 s

[a] τ_f : Fluorescence lifetime, FI : Fluorescence intensity
